# Efficient preparation of graphene oxide-immobilized copper complex and its catalytic performance in the synthesis of imidazoles

**DOI:** 10.3389/fchem.2023.1178716

**Published:** 2023-04-13

**Authors:** Shahram Heidari, Ali Zarnegaryan, Zahra Dehbanipour

**Affiliations:** Department of Chemistry, Yasouj University, Yasouj, Iran

**Keywords:** benzimidazoles, graphene oxide, heterogeneous, nanocatalyst, copper complex

## Abstract

This paper focused on the synthesis of phenylthiocarbamide-grafted graphene oxide (GO)-supported Cu complex (Cu-PTC@GO) as a highly efficient and recyclable catalyst synthesis by various analytical techniques such as TG, FT-IR, XRD, BET, N_2_ adsorption–desorption isotherms, SEM, EDX, and elemental mapping analysis. Cu-PTC@GO showed outstanding results in preparing various imidazoles with higher yields, reduced reaction time, ease of product separation, and a simple procedure. In addition, the catalyst demonstrated appreciable recyclability up to five successive runs, and there was no substantial loss in catalytic performance. The result indicated that the heterogeneous base GO catalyst performed high activity and excellent recyclability in synthesizing various imidazoles and their derivatives, owing to the unique state of the GO-supported copper complex.

## 1 Introduction

Benzimidazoles are some of the most effective heterocyclic organic compounds used as valuable intermediates in organic synthesis (Tejada-Casado et al., 2016; Li et al., 2020; Duan et al., 2020). Benzimidazole derivatives were synthesized from the condensation of aldehydes and 1,2-phenylenediamine with aromatic aldehydes under oxidative or strongly acidic conditions (Hisano et al., 1982; Czarny et al., 1996; Bello et al., 2019; Li et al., 2020). To date, various heterogeneous catalysts have been reported for benzimidazole design by utilizing the oxidative cyclization pathway (Hisano and Nanda, 2018; Marzouk et al., 2018; Shi et al., 2018; Fang et al., 2019). The heterogenization synthetic process is carried out by attaching the desired newly homogeneous catalyst to proper support *via* the non-covalent or covalent interactions (Lopez and Liu, 2020). Many efforts have been made on the heterogenization of copper catalysts on the surface of different supports, including mesoporous materials (Costa et al., 2021), functionalized polymers (Chaudhary and Sharma, 2021), resins (Xiong et al., 2021), carbon nanotubes (Şen and Gokagac, 2007; Mohd Nurazzi et al., 2021), and silica (Farhadian et al., 2021). However, few works have reported on using graphene oxide (GO) as support for the attachment of this type of catalyst and its catalytic performance (Maleki et al., 2021; Rezaei-Seresht et al., 2021; Boroumand et al., 2022; Laffafchi et al., 2022). Compared with different supports, GO is an appealing two-dimensional carbon-based material with attractive properties like mechanical stability and excellent thermal stability (Khojastehnezhad et al.,2019; Lee et al., 2021). GO possesses a unique nanostructure (just a few stacked layers or a monolayer), thermal stability, a large specific surface area, and great oxygen-carrying functionalities (Gilliland et al., 2018; Chakraborty et al., 2019). A new type of carbon nanomaterial, including a monolayer of sp^2^ carbon atoms, GO sheets are prominent due to their unique chemical properties. GO has a vast domain of applications, such as photocatalysts, catalysts, sensors, supercapacitors, wastewater treatment, energy storage, hydrogen storage, biomedical devices, and drug delivery (Daşdelen et al., 2017; Rana et al., 2019; Zarnegaryan et al., 2019; Deng et al., 2021; Kargar et al., 2021; Niakan and Masteri-Farahani, 2021; Rana et al., 2021; Xu et al., 2021; Zarnegaryan and Dehbanipour, 2021).

**SCHEME 1:**
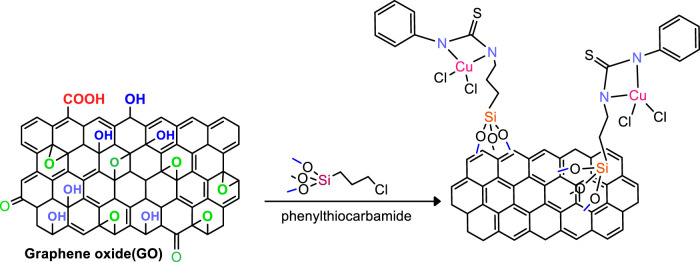
Schematic diagram of the preparation of the Cu-PTC@GO nanocatalyst.

We report herein the synthesis of a new nanocatalyst Cu-PTC@GO, where copper complexes are immobilized on a GO support (Scheme 1). The catalytic potential of the new nanocatalyst is also demonstrated, for the first time, in the synthesis of a series of pharmaceutically important benzimidazoles from aldehydes and 1,2-diaminobenzenes, under the optimized reaction conditions.

## 2 Experiments

### 2.1 Synthesis of GO-Cl

GO was prepared by a suitable modification reported by Hummers and Offerman. (1958). The chloro-functionalized GO was prepared by a slightly modified procedure (Zhang et al., 2014). In the first step, 1.0 g GO was entirely dispersed in toluene (18 mL) at 60°C. Then, (3-chloropropyl)triethoxysilane (0.5 g) was slowly added to a solution and stirred for 24 h under reflux. The GO-Cl obtained was washed with ethanol and dried under a vacuum.

### 2.2 Synthesis of PTC@GO

To immobilize a phenylthiocarbamide (PTC) ligand on GO-Cl, in a stirred solution GO-Cl (2.50 g) in CH_3_CN solvent (18 mL) at 80°C, a solution of a phenylthiocarbamide ligand (1.5 g) in ethanol (15 mL) was added dropwise. The reaction was stirred at 75°C for 24 h. Finally, the material was washed with ethanol four times and dried at 50°C overnight.

### 2.3 Synthesis of Cu-PTC@GO

To immobilize Cu metal on (PTC)@GO, a stirred solution (PTC)@GO (0.20 g) in ethanol solvent (10 mL) at 80°C, a solution of CuCl_2_ (0.1 g) in EtOH (8 mL) was added dropwise. The reaction mixture was stirred at reflux for 24 h. The nanocatalyst was then isolated by vacuum filtration. Finally, the material was washed with ethanol four times and dried at 40°C overnight. The ICP demonstrates loading of 0.52 mmol g^−1^ Cu for Cu-PTC@GO.

### 2.4 Catalytic tests

The general procedure for preparation of imidazoles: a mixture of aldehyde (1 mmol), phenylenediamine (1 mmol), and Cu-PTC@GO (12 mg containing 0.0062 mmol Cu(II)) in CH_3_CN (12 mL) was heated to 80°C. The reaction progress was monitored by thin-layer chromatography (TLC) using a mixture of ethyl acetate and hexane (1:2). During workup, the nanocatalyst was separated by filtration, and 5 mL of H_2_O and 5 mL of CH_2_Cl_2_ were added.

## 3 Results and discussion

**FIGURE 1:**
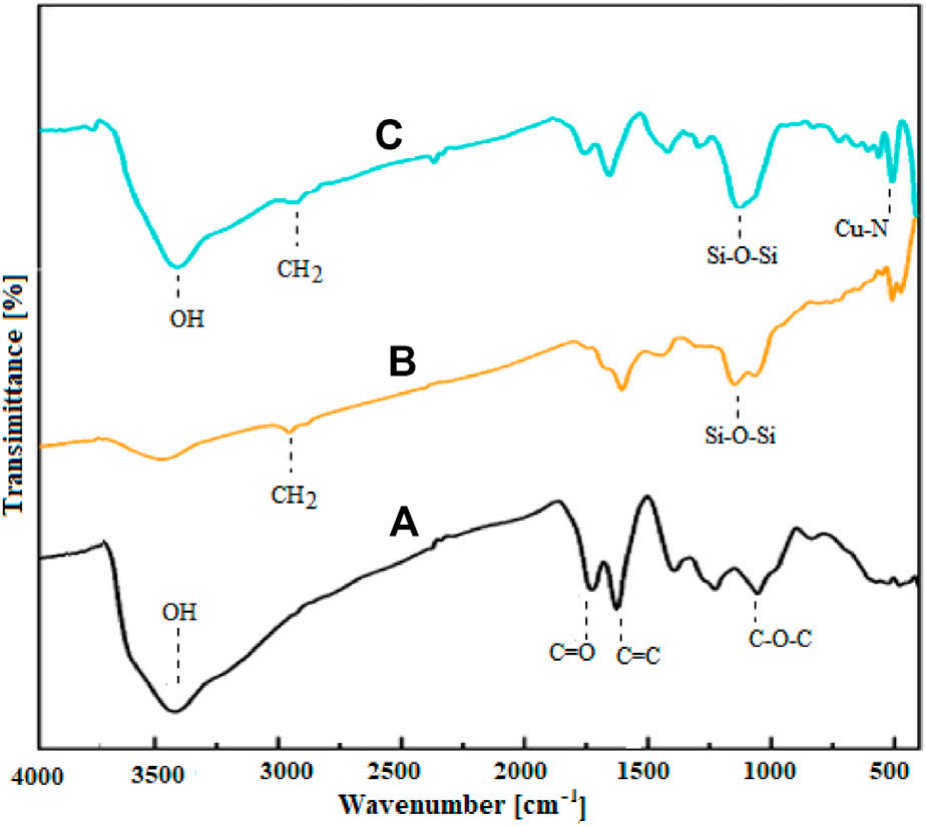
FT-IR spectra of **(A)** GO, **(B)** GO-Cl, and **(C)** Cu-PTC@GO.

In this paper, a copper phenylthiocarbamide complex was covalently grafted onto chloro-functionalized GO sheets. The FT-IR spectra of the synthesized GO, GO@ Cl, and Cu-PTC@GO catalyst are shown in Figure 1. The spectrum of GO (Figure 1A) demonstrates characteristic bands at 3382, 1717, 1621, 1223, and 1059 cm^–1^ (Figure 1A), corresponding to the presence of hydroxyl groups (O–H, H_2_O, and COOH), edge carbonyls C=O), sp^2^-hybridized aromatic compounds in a plane (C=C), and bonds of epoxides (COC and C–O), respectively (Jing et al., 2015; Ramezanzadeh et al., 2015). This issue indicates that a partial reduction process occurred by the oxidation of graphite, thus the GO fabrication. The peaks at 2839 and 2936 cm^–1^ are assigned to the vibrations of the C–H bonds of the -CH_2_ and the C–N stretching vibration (1223 cm^–1^) (Figure 1B). In addition, the observation of vibration bands 1066 and 3418 cm^–1^, assigned to Si–O–Si, and O–H vibration, respectively. The vibration band of Cu–N and Cu–S appeared at 654 and 540 cm^–1^ in the spectrum of the catalyst (Figure 1C) (Khojastehnezhad et al., 2019).

**FIGURE 2:**
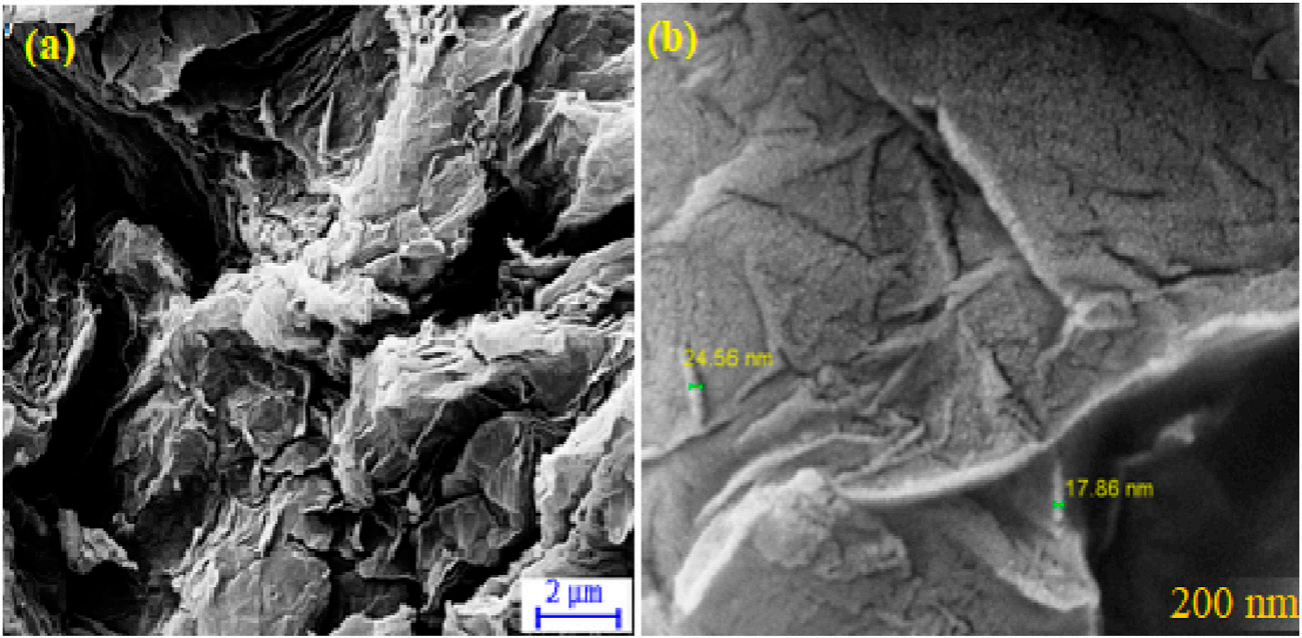
FE-SEM image of **(A)** GO and **(B)** Cu-PTC@GO.

SEM studied the surface characteristics and morphology of the synthesized GO and Cu-PTC@GO nanocatalyst; the results are illustrated in Figure 2. As shown in Figure 2A, there are large flakes of GO with macroscopic wrinkling. Compared to the GO sheets, the FE-SEM image of Cu-PTC@GO (Figure 2B) exhibited that the nanocatalyst roughly comprises a wrinkled layered structure.

**FIGURE 3:**
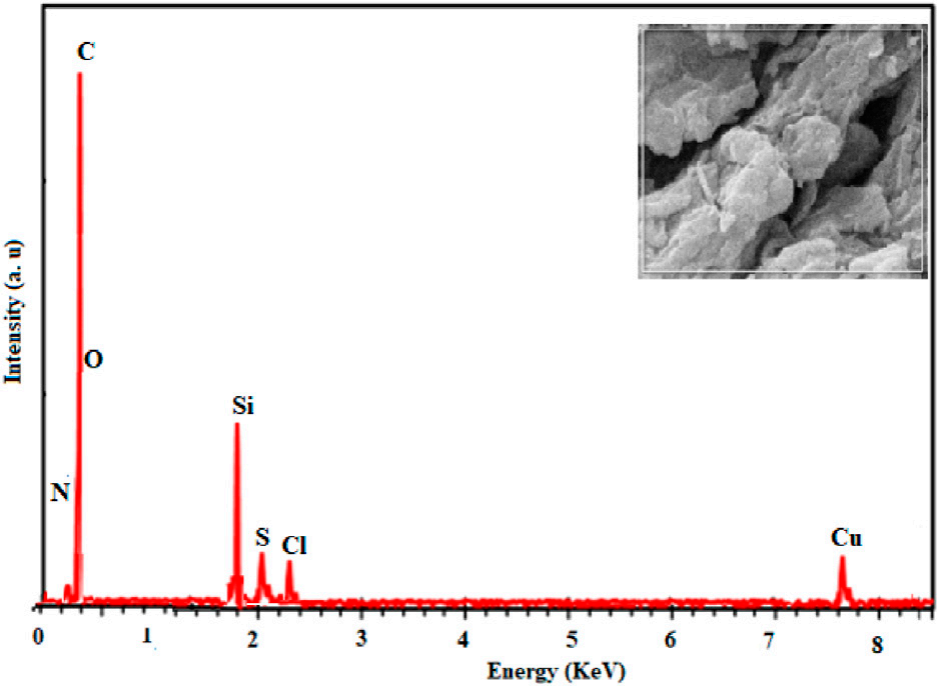
EDX analysis of Cu-PTC@GO.

Figure 3 shows the corresponding EDX spectrum of Cu-PTC@GO (Figure 3). The presence of Cu, O, Si, Cl, S, and N in the texture of Cu-PTC@GO was confirmed by the EDX spectrum.

**FIGURE 4:**
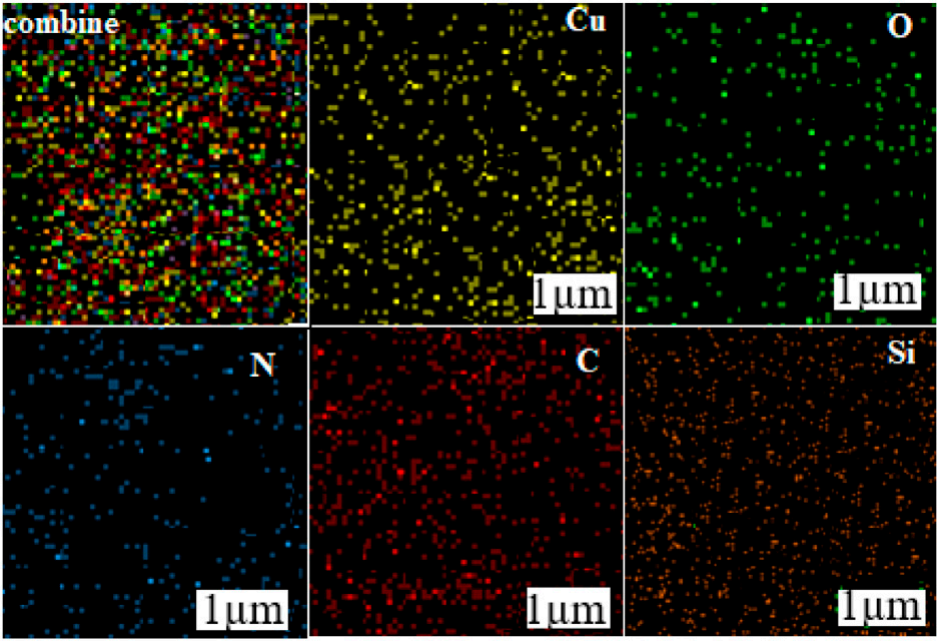
SEM-EDX element mapping of Cu-PTC@GO.

Figure 4 represents the element mapping images of the Cu-PTC@GO catalyst surface, with different colors indicating the presence of various elements after supporting Cu (PTC) onto surface GO.

**FIGURE 5:**
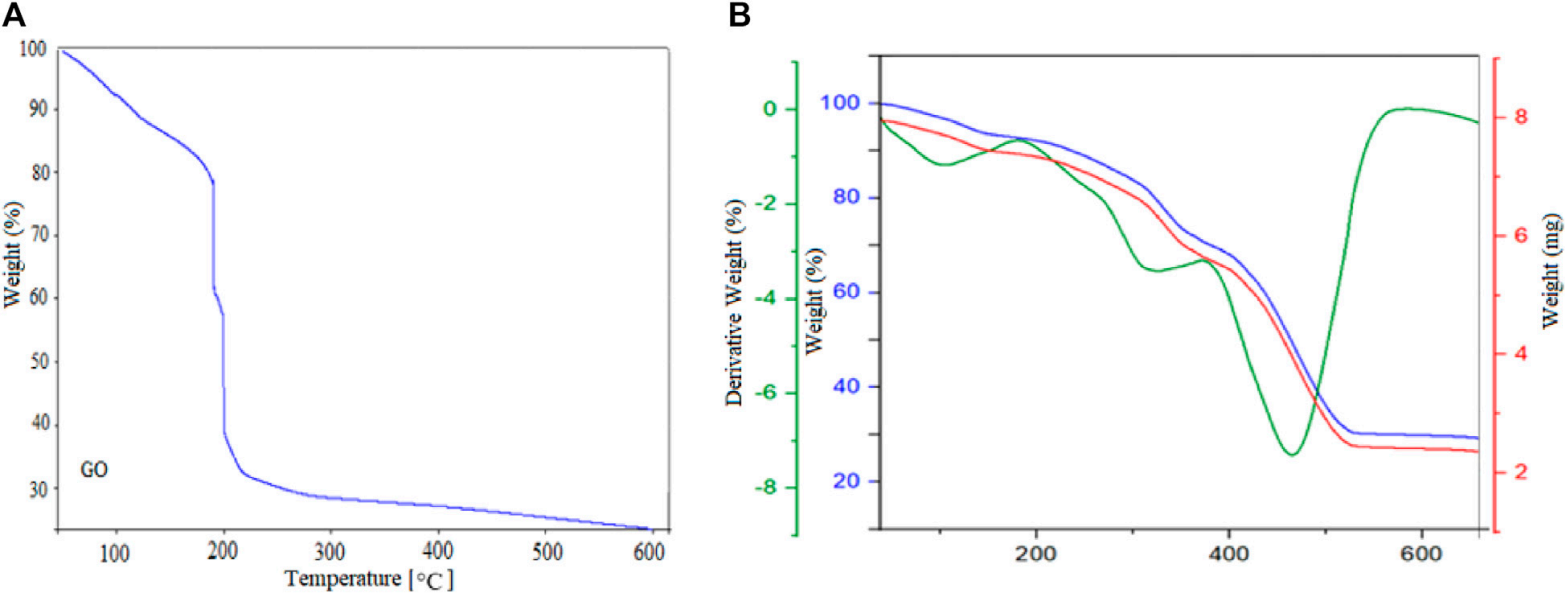
TGA curves of **(A)** GO and **(B)** Cu-PTC@GO.

Figure 5 represents the thermal gravimetric analysis (TGA) of the GO and Cu-PTC@GO nanocatalyst. The TGA analysis was performed using the heating rate of 10°C min^−1^ from 25°C to 600°C in an argon flow at a rate of 100 mL min^−1^. The weight loss (Figures 5A, B) for GO and Cu-PTC@GO below 140°C is assigned to decomposing oxygen-carrying functionalities and evaporation adsorbed water (Li et al., 2014a; Du et al., 2018). As the temperature increased, the disintegration of GO appeared from 150°C to 350°C, resulting from the pyrolysis of the oxygen involving functions such as epoxy, carboxyl, and hydroxyl groups (Zhang et al., 2009; Wu et al., 2012). The other two weight loss processes (Figure 5B) observed in the temperature at around 230°C–384°C and 385°C–490°C are mainly related to the degradation of the grafted urethane chains, respectively (Kumar et al., 2019).

**FIGURE 6:**
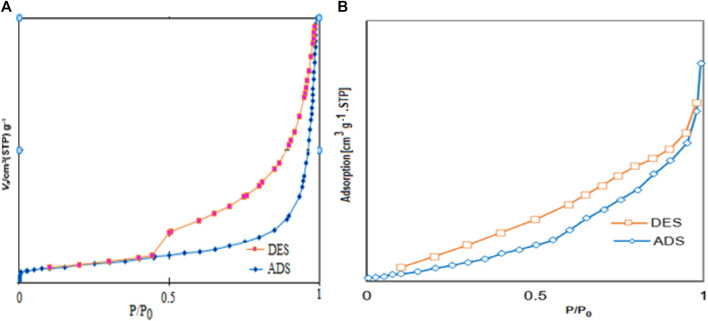
Nitrogen adsorption–desorption isotherms of **(A)** GO and **(B)** Cu-PTC@GO.

N_2_ physisorption experiment results of GO and the Cu-PTC@GO nanocatalyst are shown in Figure 6. The specific surface area of GO before modification was 84.73 m^2^g^−1^, and the average pore diameter was 26.53 nm.

The isotherms of the Cu-PTC@GO nanocatalyst match well with the type V BET catalysts with hysteresis loops in the relative pressure range of 0.2–1.0. The surface area was 28.4 m^2^ g^−1^ for the Cu-PTC@GO nanocatalyst.

**FIGURE 7:**
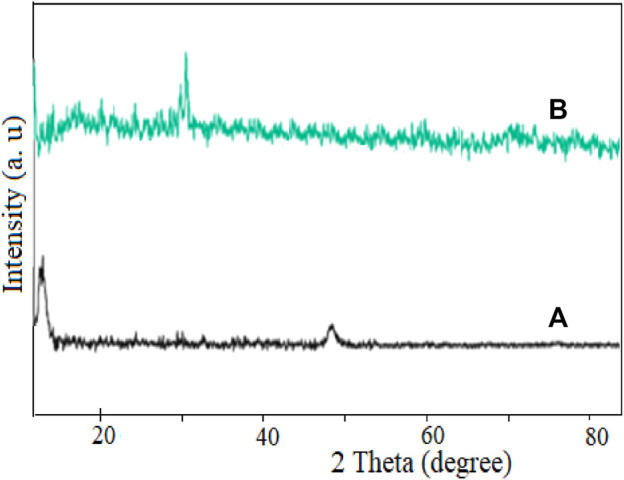
XRD patterns of **(A)** GO and **(B)** Cu-PTC@GO.

X-ray diffraction (XRD) patterns of GO and Cu-PTC@GO are exhibited in Figure 7. In the primary GO (Figure 7A), the peak at 2θ = 11.1° belongs to the (001) reflection of graphene oxide (Li et al., 2014b; Zhang, et al., 2009). In contrast, the XRD powder patterns of Cu-PTC@GO (Figure 7B) do not present a peak characteristic of GO, while a broad band centered at ca. 2θ = 30.58° is observed. However, for Cu-PTC@GO, the peaks shifted to lower angles, owing to the support of the copper complex and increasing the spacing between the nanosheets.

### 3.1 Catalytic tests of Cu-PTC@GO

To investigate the catalytic activity of the synthesized Cu-PTC@GO catalyst, after wide characterization by different techniques, it was evaluated as a catalyst for the fabrication of imidazole. Afterward, various factors were investigated to achieve the standard condition for this reaction. The catalytic activity was explored with phenylenediamine (1 mmol), benzaldehyde (1 mmol), and Cu-PTC@GO (12 mg) in CH_3_CN as the solvent under reaction conditions (Table 1). Regarding the solvent effect, the model reaction employed various solvents, ethanol, toluene, acetonitrile, methanol, tetrahydrofuran, n-hexane, and dichloromethane ethyl acetate, and solvent-free condition with Cu-PTC@GO (12 mg) (entries 1–8). Among different solvents, CH_3_CN solvent gave the highest yield. We have tested the effect of a catalyst using CuCl_2_.2H_2_O, PTC@GO, and Cu-PTC@GO (Table 1, entries 8–10).

**TABLE 1:** Optimization of reaction condition.

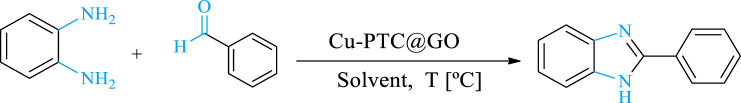					
Enter	Solvent	Name of the catalyst	T [°C]	Catalyst [ mg]	Yield [%]
1	CH_3_CN	Cu-PTC@GO	80	12	98
2	Toluene	Cu-PTC@GO	80	12	67
3	EtOH	Cu-PTC@GO	80	12	57
4	CH_3_OH	Cu-PTC@GO	80	12	53
5	EtOAc	Cu-PTC@GO	80	12	48
6	Solvent-free	Cu-PTC@GO	80	12	-
7	CH_2_Cl_2_	Cu-PTC@GO	80	12	71
8	THF	Cu-PTC@GO	80	12	79
9	CH_3_CN	PTC@GO	80	12	43
10	CH_3_CN	CuCl_2_.2H_2_O	80	12	65
11	CH_3_CN	Cu-PTC@GO	80	6	78
12	CH_3_CN	Cu-PTC@GO	80	18	98
13	CH_3_CN	Cu-PTC@GO	60	12	82
14	CH_3_CN	Cu-PTC@GO	40	12	71
15	CH_3_CN	Cu-PTC@GO	85	12	98

The results showed that the Cu-PTC@GO nanocatalyst furnished a 98% yield after 2 h. The nanocatalyst loading of Cu-PTC@GO (entries 11 and 12) was also analyzed. Interestingly, the catalytic activities of Cu-PTC@GO were found to be maximum at 12 mg of the nanocatalyst at 80°C. Furthermore, the temperature was evaluated and the model reaction was carried out at 40°C–85°C (entries 13–15). The yield remained constant when the temperature decreased below 80°C (entries 13 and 14) and increased above 80°C (entry 15).

With the stipulated optimized reaction conditions, the substrate scope for the catalyst is explored for the diverse range of several aromatic and aliphatic aldehydes with phenylenediamine (Table 2). Aliphatic aldehydes took a longer reaction time and provided moderate yields compared to aromatic aldehydes. The aromatic aldehydes produced the corresponding products in better yields than the aliphatic aldehydes. Aromatic aldehydes involving the electron-donating group yielded better than the electron-withdrawing group.

**TABLE 2:** Investigation of the substrate scope.^a^

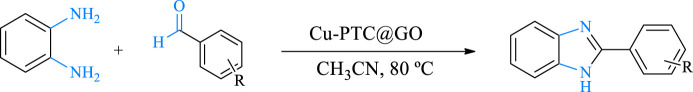					
Entry	Aldehyde	Benzimidazole	Time [h]	Yield [%]	Found MP [°C]
1	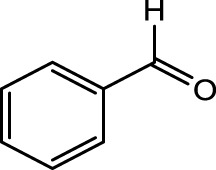	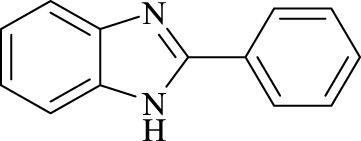	2	98	293–295
2	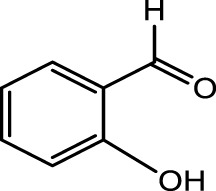	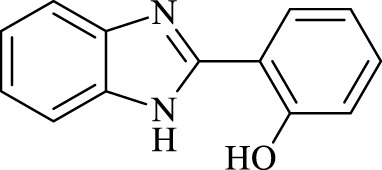	4	89	238–240
3	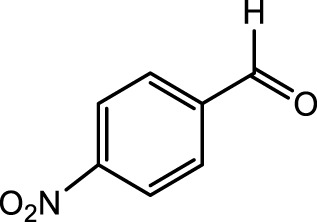	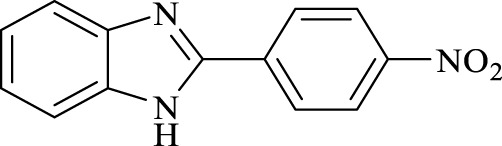	3	87	319–321
4	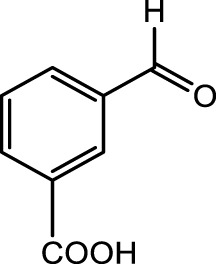	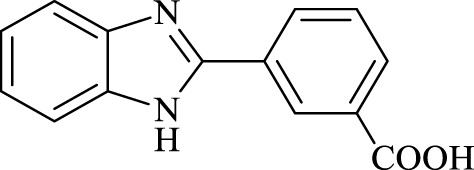	3.5	86	>300
5	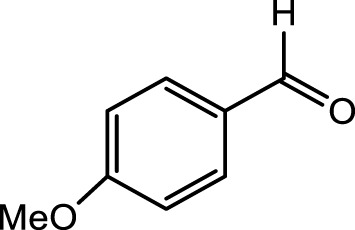	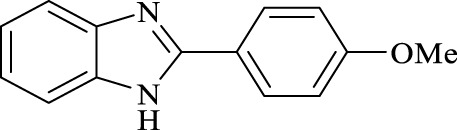	3	81	293–296
6	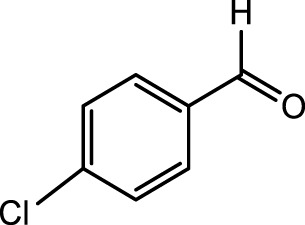	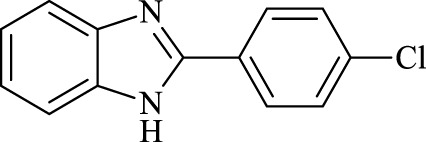	2.5	85	291–292
7	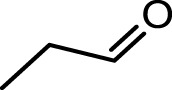	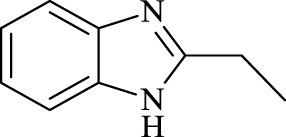	5	73	172–174
8	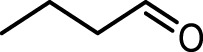	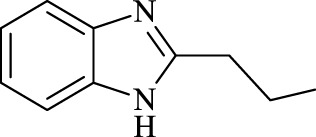	6.5	77	154–156

^a^ Conditions: catalyst (12 mg), aldehyde (1 mmol), phenylenediamine (1 mmol), CH_3_CN (12 mL), and 80°C.

### 3.2 Catalyst reusability and stability

**FIGURE 8:**
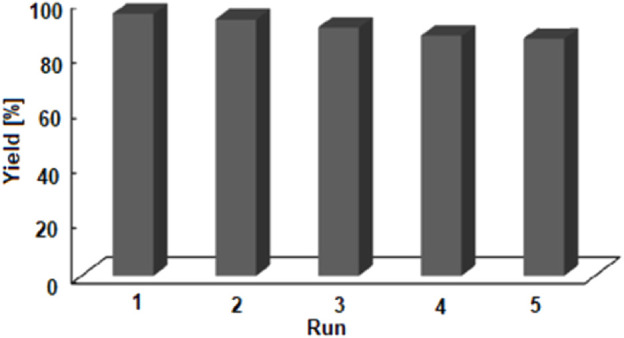
Recycling experiments of Cu-PTC@GO.

The reusability of a nanocatalyst is important from economic and environmental points of view. Therefore, the reusability and recyclability of Cu-PTC@GO were investigated for synthesizing benzimidazoles under optimized conditions (CH_3_CN = 12 mL, T = 80°C, catalyst = 0.05 mol%, benzaldehyde = 1 mmol, and phenylenediamine = 1 mmol). Subsequently, after each run, the nanocatalyst was recovered by centrifugation, washed with CH_3_CN, and dried at 60°C. The recovered nanocatalyst was utilized in another experiment to estimate its reusability. As shown in Figure 8, no noticeable change in the catalytic activity of Cu-PTC@GO was observed even after five cycles. The leach metal in the filtrate was determined by ICP analysis and found to be 0.14 ppm Cu.

### 3.3 Hot filtration test

**FIGURE 9:**
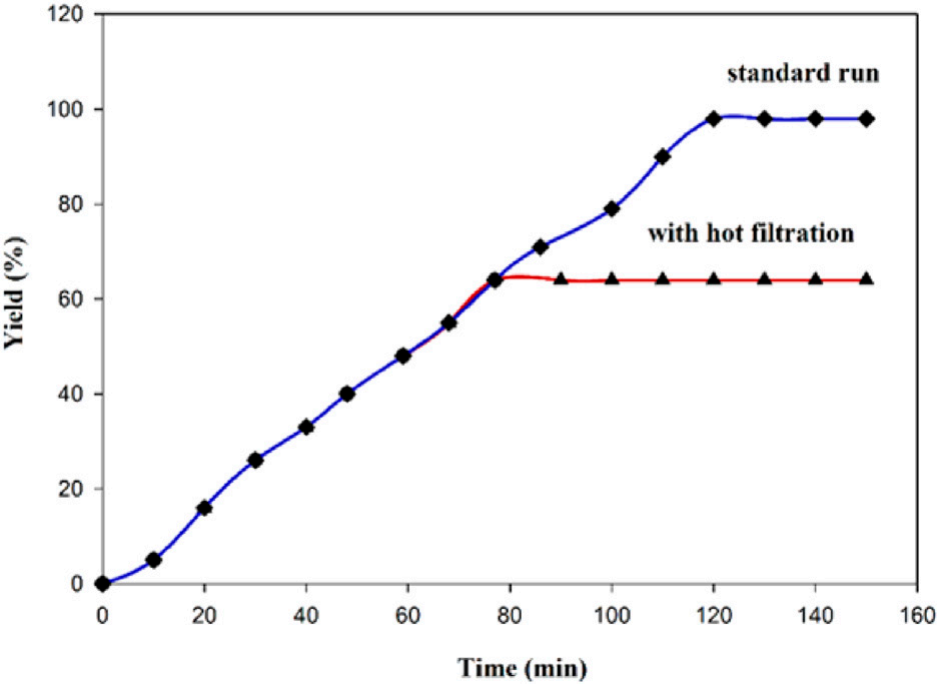
Hot filtration test of benzimidazoles.

To investigate the heterogeneous nature of the catalyst, a hot filtration test was conducted for benzimidazoles under the optimized reaction conditions in the presence of a heterogeneous catalyst (Figure 9). After 1 h, the reaction was paused and the catalyst was separated through filtration from the reaction mixture under hot conditions, which resulted in a 65% yield of the product. The filtrate was again transferred into a reaction flask and heated at 80°C for 3 h. No enhancement in the yield of the product could be obtained after the removal of the catalyst from the reaction mixture, which confirmed the heterogeneous nature of the catalyst. The results obtained from ICP showed that no significant amounts of Cu are leached during the reaction.

The results obtained for the preparation of imidazole were compared with the previously reported procedures to show the performance of the Cu-PTC@GO catalyst (Table 3). As a result, it showed that this Cu-PTC@GO catalyst has better activity in reaction time and product yield.

**TABLE 3:** Comparison catalytic activity of Cu-PTC@GO with similar systems.

Entry	Name of the catalyst	Conditions	Yield [%]	Reference
1	Cu-PTC@GO	CH_3_CN/65°C/2 h	98	This work
2	Fe_3_O_4_–CB	tBuOK/120°C/8 h	88	Verma et al. (2022)
3	GO	CH_3_OH/65°C/4 h	81	Dhopte et al. (2016)
4	A-FGO	THF/reflux/2 h	86	Hanoon et al. (2017)
5	CoCl_2_.6H_2_O	CH_3_OH/RT/4 h	81	Khan et al. (2009)
6	HNO_3_@nano SiO_2_	Solvent-free/90°C/2.5 h	85	Nikoofar and Dizgarani (2018)

**SCHEME 2:**
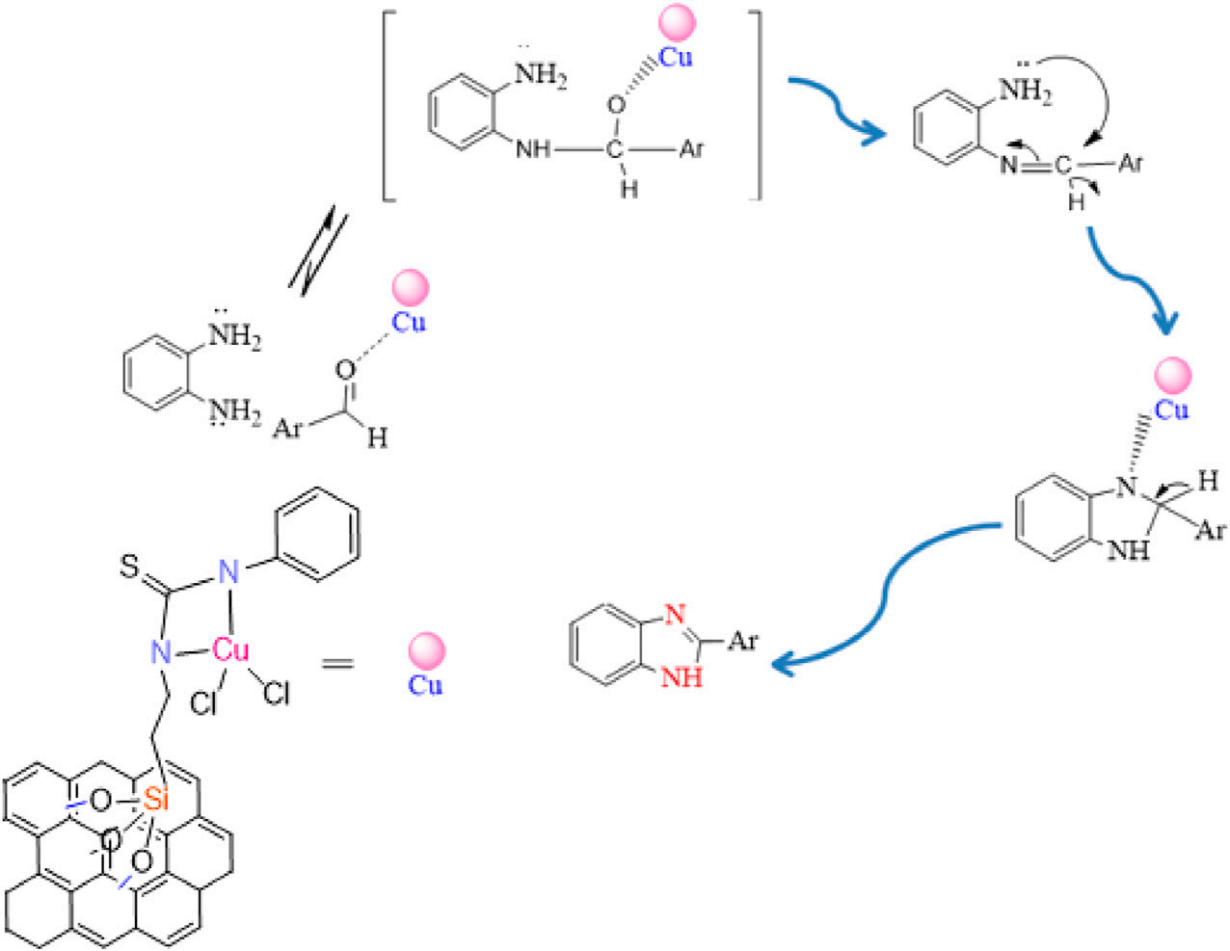
Plausible mechanism pathway for the synthesis of benzimidazole.

A mechanism was introduced to prepare the imidazole heterocyclic compound from phenylenediamine and aromatic aldehydes using the Cu-PTC@GO catalyst (Scheme 2). The reaction between an amine and an aldehyde proceeds *via* the condensation reaction mechanism, which forms a Schiff base and includes the oxidative cyclization reaction (Verma et al., 2022). The activation of the ring aldehyde on the GO surface enhances the electrophilicity of the C=O group, thereby facilitating the formation of imine. Thus, the ring closure reaction occurs *via* the nucleophilic attack between the substituted amine and imine intermediate. Finally, the desired heterocyclic product was achieved.

## 4 Conclusion

The GO was employed as a support for preparing the copper complex. Different characterization methods characterized the properties of the prepared catalyst. The catalytic efficiency of the catalyst was evaluated in the synthesis of imidazole derivatives. The heterogonous catalyst was used for the synthesis of imidazoles with excellent yields. This catalyst was employed for five runs without losing activity, demonstrating its recoverability, easy separation, reusability, and efficiency. These features make this nanocatalyst an appropriate heterogeneous catalyst for synthesizing imidazoles.

## Data Availability

The raw data supporting the conclusion of this article will be made available by the authors, without undue reservation.

## References

[B1] Bello A. U. Uzairu A. Shallangwa G. A. (2019). Prediction of inhibition performance of some benzimidazole derivatives against steel corrosion through QSAR and molecular dynamic simulation. J. Mat. Environ. Sci. 10, 1–14.

[B2] Boroumand H. Alinezhad H. Maleki B. Peiman S. (2022). Triethylenetetramine-grafted magnetic graphene oxide (Fe_3_O_4_@ GO-NH_2_) as a reusable heterogeneous catalyst for the one-pot synthesis of 2-amino-4 H-benzopyran derivatives. Polycycl. Aromat. Compd. 2022, 1–17. 10.1080/10406638.2022.2140683

[B3] Chakraborty T. Sarkar A. Chattopadhyay T. (2019). Pd (0) immobilized on Fe_3_O_4_@ AHBA: An efficient magnetically separable heterogeneous nanocatalyst for C–C coupling reactions. J. Coord. Chem. 72, 3430–3443. 10.1080/00958972.2019.1687890

[B4] Chaudhary V. Sharma S. (2021). Study of ethylbenzene oxidation over polymer-silica hybrid supported Co (II) and Cu (II) complexes. Catal. Today. 375, 601–613. 10.1016/j.cattod.2020.02.043

[B5] Costa J. A. S. de Jesus R. A. Santos D. O. Neris J. B. Figueiredo R. T. Paranhos C. M. (2021). Synthesis, functionalization, and environmental application of silica-based mesoporous materials of the M41S and SBA-n families: A review. J. Environ. Chem. Eng. 9, 105259. 10.1016/j.jece.2021.105259

[B6] Czarny A. Wilson W. D. Boykin D. W. (1996). Synthesis of mono‐cationic and dicationic analogs of hoechst 33258. J. Heterocycl. Chem. 33, 1393–1397. 10.1002/jhet.5570330463

[B7] Daşdelen Z. Yıldız Y. Eriş S. Şen F. (2017). Enhanced electrocatalytic activity and durability of Pt nanoparticles decorated on GO-PVP hybride material for methanol oxidation reaction. Appl. Catal. B Environ. 219, 511–516. 10.1016/j.apcatb.2017.08.014

[B8] Deng H. Yin J. Ma J. Zhou J. Zhang L. Gao L. (2021). Exploring the enhanced catalytic performance on nitro dyes via a novel template of flake-network Ni-Ti LDH/GO *in-situ* deposited with Ag_3_PO_4_ NPs. Appl. Surf. Sci. 543, 148821. 10.1016/j.apsusc.2020.148821

[B9] Dhopte K. B. Zambare R. S. Patwardhan A. V. Nemade P. R. (2016). Role of graphene oxide as a heterogeneous acid catalyst and benign oxidant for synthesis of benzimidazoles and benzothiazoles. RSC Adv. 6, 8164–8172. 10.1039/c5ra19066e

[B10] Du W. Zhang Z. Fan W. Gao W. Su H. Li Z. (2018). Fabrication and evaluation of polydimethylsiloxane modified gelatin/silicone rubber asymmetric bilayer membrane with porous structure. Mat. Des. 158, 28–38. 10.1016/j.matdes.2018.08.017

[B11] Duan Z. Zhang L. Zhang W. Lu L. Zeng L. Shi R. (2020). Palladium-catalyzed electro-oxidative C–H amination toward the synthesis of pyrido [1, 2-a] benzimidazoles with hydrogen evolution. ACS Catal. 10, 3828–3831. 10.1021/acscatal.0c00103

[B12] Fang S. Yu H. Yang X. Li J. Shao L. (2019). Nickel‐Catalyzed construction of 2, 4‐disubstituted imidazoles via C–C coupling and C− N condensation cascade reactions. Adv. Synth.Catal. 361, 3312–3317. 10.1002/adsc.201900096

[B13] Farhadian N. Liu S. Asadi A. Shahlaei M. Moradi S. (2021). Enhanced heterogeneous fenton oxidation of organic pollutant via Fe-containing mesoporous silica composites: A review. Moradi, J. Mol. Liq. 321, 114896. 10.1016/j.molliq.2020.114896

[B14] Gilliland S. E. III Tengco J. M. M. Yang Y. Regalbuto J. R. Castano C. E. Gupton B. F. (2018). Electrostatic adsorption-microwave synthesis of palladium nanoparticles on graphene for improved cross-coupling activity. Appl. Catal. A General 550, 168–175. 10.1016/j.apcata.2017.11.007

[B15] Hanoon H. D. Kowsari E. Abdouss M. Zandi H. Ghasemi M. H. (2017). Efficient preparation of acidic ionic liquid-functionalized reduced graphene oxide and its catalytic performance in synthesis of benzimidazole derivatives. Res. Chem. Intermed. 43, 1751–1766. 10.1007/s11164-016-2727-0

[B16] Hisano T. Ichikawa M. Tsumoto K. Tasaki M. (1982). Synthesis of benzoxazoles, benzothiazoles and benzimidazoles and evaluation of their antifungal, insecticidal and herbicidal activities. Chem. Pharm. Bull. 30, 2996–3004. 10.1248/cpb.30.2996

[B17] Hossain M. Nanda A. K. (2018). A review on heterocyclic: Synthesis and their application in medicinal chemistry of imidazole moiety. Science 6, 83–94. 10.11648/j.sjc.20180605.12

[B18] Hummers W. S. Offeman R. E. (1958). Preparation of graphitic oxide. J. Am. Chem. Soc. 80, 1339. 10.1021/ja01539a017

[B19] Jing Q. Liu W. Pan Y. Silberschmidt V. V. Li L. Dong Z. (2015). Chemical functionalization of graphene oxide for improving mechanical and thermal properties of polyurethane composites. Dong, Mat. Des. 85, 808–814. 10.1016/j.matdes.2015.07.101

[B20] Kargar S. Elhamifar D. Zarnegaryan A. (2021). Ionic liquid modified graphene oxide supported Mo-complex: A novel, efficient and highly stable catalyst. Surf. Interfaces 23, 100946. 10.1016/j.surfin.2021.100946

[B21] Khan A. T. Parvin T. Choudhury L. H. (2009). A simple and convenient one-pot synthesis of benzimidazole derivatives using cobalt (II) chloride hexahydrate as catalyst. Synth. Commun. 39, 2339–2346. 10.1080/00397910802654815

[B22] Khojastehnezhad A. Bakavoli M. Javid A. Khakzad Siuki M. M. Moeinpour F. (2019). Covalently copper (II) porphyrin cross-linked graphene oxide: Preparation and catalytic activity. Catal. Lett. 149, 713–722. 10.1007/s10562-019-02665-2

[B23] Kumar A. Layek S. Agrahari B. Kujur S. Pathak D. D. (2019). Graphene oxide immobilized copper (II) Schiff base complex [GO@ AF‐SB‐Cu]: A versatile catalyst for chan‐lam coupling reaction. ChemistrySelect 4, 1337–1345. 10.1002/slct.201803113

[B24] Laffafchi F. Tajbakhsh M. Sarrafi Y. Maleki B. Ghani M. (2022). Cu-modified magnetic creatine as an efficient catalyst for regioselective preparation of 1, 2, 3-triazoles derivatives. Taylor & Francis, Polycyclic Aromatic Compounds, 1–17.

[B25] Lee S. J. Theerthagiri J. Nithyadharseni P. Arunachalam P. Balaji D. Kumar A. M. (2021). Heteroatom-doped graphene-based materials for sustainable energy applications: A review. Sust. Energy Rev. 143, 110849. 10.1016/j.rser.2021.110849

[B26] Li B. Tayebee R. Esmaeili E. Namaghi M. S. Maleki B. (2020a). Selective photocatalytic oxidation of aromatic alcohols to aldehydes with air by magnetic WO_3_ZnO/Fe_3_O_4_. *in situ* photochemical synthesis of 2-substituted benzimidazoles. RSC Adv. 10, 40725–40738. 10.1039/d0ra08403d 35519184 PMC9057692

[B27] Li S. Liang Q. Ahmed S. A. H. Zhang J. (2020b). Simultaneous determination of five benzimidazoles in agricultural foods by core-shell magnetic covalent organic framework nanoparticle–based solid-phase extraction coupled with high-performance liquid chromatography. Food Anal. Methods 13, 1111–1118. 10.1007/s12161-020-01708-4

[B28] Li Z. Wu S. Zheng D. Ding H. Wang X. Yang X. (2014a). Enhanced aerobic epoxidation of styrene with copper (II), cobalt (II), iron (III), or oxovanadium (IV) salen complexes immobilized onto carbon‐coated Fe_3_O_4_ nanoparticles hybridized with graphene sheets. ChemPlusChem 79, 716–724. 10.1002/cplu.201300424

[B29] Li Z. Wu S. Zheng D. Liu J. Liu H. Lu H. (2014b). Dioxomolybdenum(VI) complex covalently attached to amino-modified graphene oxide: Heterogeneous catalyst for the epoxidation of alkenes. Appl. Organometal. Chem. 28, 317–323. 10.1002/aoc.3127

[B30] Lopez A. Liu J. (2020). Covalent and noncovalent functionalization of graphene oxide with DNA for smart sensing. Adv. Intell. Syst. 2, 2000123. 10.1002/aisy.202000123

[B31] Maleki B. Taheri F. Tayebee R. Adibian F. (2021). Dendrimer-functionalized magnetic graphene oxide for knoevenagel condensation. Org. Prep. Proced. Int. 53, 284–290. 10.1080/00304948.2021.1875799

[B32] Marzouk A. A. Abu‐Dief A. M. Abdelhamid A. A. (2018). Hydrothermal preparation and characterization of ZnFe_2_O_4_ magnetic nanoparticles as an efficient heterogeneous catalyst for the synthesis of multi‐substituted imidazoles and study of their anti‐inflammatory activity. Appl. Organomet. Chem. 32, e3794. 10.1002/aoc.3794

[B33] Mohd Nurazzi N. Asyraf M. M. Khalina A. Abdullah N. Sabaruddin F. A. Kamarudin S. H. (2021). Fabrication, functionalization, and application of carbon nanotube-reinforced polymer composite: An overview. Polymers 13, 1047. 10.3390/polym13071047 33810584 PMC8037012

[B34] Niakan M. Masteri-Farahani M. (2022). Ultrafine and well-dispersed Pd-Ni bimetallic catalyst stabilized by dendrimer-grafted magnetic graphene oxide for selective reduction of toxic nitroarenes under mild conditions. J. Hazard. Mat. 424, 127717. 10.1016/j.jhazmat.2021.127717 34799155

[B35] Nikoofar K. Dizgarani S. M. (2018). HNO_3_ immobilized on nano SiO_2_: A novel efficient heterogeneous catalytic system for the synthesis of 2-substituted oxazolines, imidazolines, thiazolines, and 2-aryl-1H-benzimidazoles under solvent-free conditions. Bulg. Chem. Commun. 50, 100–110.

[B51] Ramezanzadeh B. Ghasemi E. Mahdavian M. Changizi E. Moghadam M. M. (2015). Characterization of covalently-grafted polyisocyanate chains onto graphene oxide for polyurethane composites with improved mechanical properties. Chem. Eng. J. 281, 869–883.

[B49] Rana S. Varadwaj G. B. B. Jonnalagadda S. B. (2019). A facile synthesis of molybdenum promoted reduced graphene oxide as catalyst towards epoxidation of cyclohexene. ChemistrySelect 4, 5381–5385.

[B36] Rana S. Varadwaj G. B. B. Jonnalagadda S. B. (2021). Manganese oxide supported partially reduced graphene oxide as a highly active and durable catalyst for the amination of benzene. Catal. Commun. 157, 106329. 10.1016/j.catcom.2021.106329

[B37] Rezaei-Seresht E. Bakhshi-Noroozi M. Maleki B. (2021). Piperazine-grafted magnetic reduced graphene oxide (Fe_3_O_4_@ rGO-NH) as a reusable heterogeneous catalyst for gewald three-component reaction. Polycycl. Aromat. Compd. 41 (9), 1944–1952. 10.1080/10406638.2019.1708417

[B38] Şen F. Gökaǧaç G. (2007). Activity of carbon-supported platinum nanoparticles toward methanol oxidation reaction: Role of metal precursor and a new surfactant, tert-octanethiol. J. Phys. Chem. C 111, 1467–1473. 10.1021/jp065809y

[B39] Shi S. Xu K. Jiang C. Ding Z. (2018). ZnCl_2_-Catalyzed [3+ 2] cycloaddition of benzimidates and 2 H-azirines for the synthesis of imidazoles. J. Org. Chem. 83, 14791–14796. 10.1021/acs.joc.8b02437 30394746

[B40] Tejada-Casado C. Hernández-Mesa M. del Olmo-Iruela M. García-Campaña A. M. (2016). Capillary electrochromatography coupled with dispersive liquid-liquid microextraction for the analysis of benzimidazole residues in water samples. Talanta 161, 8–14. 10.1016/j.talanta.2016.08.012 27769484

[B41] Verma S. Kujur S. Sharma R. Pathak D. D. (2022). Cucurbit [6] uril-supported Fe_3_O_4_ magnetic nanoparticles catalyzed green and sustainable synthesis of 2-substituted benzimidazoles via acceptorless dehydrogenative coupling. ACS omega 7, 9754–9764. 10.1021/acsomega.1c07350 35350370 PMC8945128

[B42] Wu C. Huang X. Wang G. Wu X. Yang K. Li S. (2012). Hyperbranched-polymer functionalization of graphene sheets for enhanced mechanical and dielectric properties of polyurethane composites. J. Mat. Chem. A 22, 7010–7019. 10.1039/c2jm16901k

[B43] Xiong W. Zhang P. Liu S. Lv Y. Zhang D. (2021). Catalyst-free synthesis of phenolic-resin-based carbon nanospheres for simultaneous electrochemical detection of Cu (II) and Hg (II). Diam. Relat. Mat. 111, 108170. 10.1016/j.diamond.2020.108170

[B44] Xu Y. Wang R. Wang J. Li J. Jiao T. Liu Z. (2021). Facile fabrication of molybdenum compounds (Mo_2_C, MoP and MoS_2_) nanoclusters supported on N-doped reduced graphene oxide for highly efficient hydrogen evolution reaction over broad pH range. Chem. Eng. J. 417, 129233. 10.1016/j.cej.2021.129233

[B46] Zarnegaryan A. Dehbanipour Z. (2021). Iron (II) complex supported on graphene nanosheet: An efficient and heterogeneous catalyst for epoxidation of alkenes. Appl. Surf. Sci. Adv. 4, 100074. 10.1016/j.apsadv.2021.100074

[B50] Zarnegaryan A. Pahlevanneshan Z. Moghadam M. Tangestaninejad S. Mirkhani V. Mohammdpoor-Baltork I. (2019). Copper (II) Schiff base complex immobilized on graphene nanosheets: A heterogeneous catalyst for epoxidation of olefins. J. Iran. Chem. Soc. 16, 747–756.

[B47] Zhang D. D. Zu S. Z. Han B. H. (2009). Inorganic–organic hybrid porous materials based on graphite oxide sheets. Carbon 47, 2993–3000. 10.1016/j.carbon.2009.06.052

[B48] Zhang W. Zhao Q. Liu T. Gao Y. Li Y. Zhang G. (2014). Phosphotungstic acid immobilized on amine-grafted graphene oxide as acid/base bifunctional catalyst for one-pot tandem reaction. Ind. Eng. Chem. Res. 53, 1437–1441. 10.1021/ie403393u

